# Propolis Extract and Chitosan Improve Health of *Nosema ceranae* Infected Giant Honey Bees, *Apis dorsata* Fabricius, 1793

**DOI:** 10.3390/pathogens10070785

**Published:** 2021-06-22

**Authors:** Sanchai Naree, Rujira Ponkit, Evada Chotiaroonrat, Christopher L. Mayack, Guntima Suwannapong

**Affiliations:** 1 Biological Science Program, Faculty of Science, Burapha University, Chon Buri 20131, Thailand; 60810001@go.buu.ac.th (S.N.); 59810030@go.buu.ac.th (R.P.); 62810007@go.buu.ac.th (E.C.); 2 Molecular Biology, Genetics, and Bioengineering, Faculty of Engineering and Natural Sciences, Sabanci University, 34956 Istanbul, Turkey; christopher.mayack@sabanciuniv.edu

**Keywords:** *Apis dorsata*, chito-oligosaccharide, *Nosema ceranae*, propolis

## Abstract

*Nosema ceranae* is a large contributing factor to the most recent decline in honey bee health worldwide. Developing new alternative treatments against *N. ceranae* is particularly pressing because there are few treatment options available and therefore the risk of increased antibiotic resistance is quite high. Recently, natural products have demonstrated to be a promising avenue for finding new effective treatments against *N. ceranae*. We evaluated the effects of propolis extract of stingless bee, *Tetrigona apicalis* and chito-oligosaccharide (COS) on giant honey bees, *Apis dorsata*, experimentally infected with *N. ceranae* to determine if these treatments could improve the health of the infected individuals. Newly emerged *Nosema*-free bees were individually inoculated with 10^6^*N. ceranae* spores per bee. We fed infected and control bees the following treatments consisting of 0%, 50%, propolis extracts, 0 ppm and 0.5 ppm COS in honey solution (*w/v*). Propolis extracts and COS caused a significant increase in trehalose levels in hemolymph, protein contents, survival rates and acini diameters of the hypopharyngeal glands in infected bees. Our results suggest that propolis and COS could improve the health of infected bees. Further research is needed to determine the underlying mechanisms responsible for the improved health of the infected bees.

## 1. Introduction

Honey bees play a vital role in agricultural crop production and ecosystem stability due to their pollination services [1,2,3,4,5,6]. Despite their importance there has been a global decline in bee health around the world at unsustainable rates [7,8,9]. The health decline can be attributed to a number of health factors such as pesticide exposure and poor nutrition, with parasitic infections as one of the major contributors [10,11]. *Nosema* disease or nosemosis is one of the most widespread parasitic infections of adult honey bees and has been implicated to play a major role in the most recent global bee health decline [12,13,14]. Nosemosis is caused by three species of microsporidia, *Nosema apis*, *N. ceranae* and *N. neumanni*, but the most prevalent strain found in honey bees that has emerged is *N. ceranae* displacing much of the *N. apis* infections worldwide [15,16]. Nosemosis is considered to be a chronic infection that does not exhibit obvious external disease symptoms, but can cause a poor nutrient and energy absorption leading to a suppressed immune function and ultimately a shortened life span [17,18,19,20,21]. Infected bees have evidence of lower trehalose and lipid levels, and a reduced hypopharyngeal gland resulting from the poor nutrient absorption across the gut lining [22,23,24]. *N. ceranae* primarily lives and reproduces in the gut lining which is likely the cause for the poor nutrient absorption in infected bees [25,26]. Consequently, infected individuals suffer from energetic stress, which results in increased bee mortality on the individual and colony level [24,27,28,29,30].

There are only a few treatment options on the market for controlling Nosemosis. The antibiotic Fumagillin has been on the market for a long time, but it is unable to kill the mature spore form of the parasite [31], so reinfections can occur [32,33]. Moreover, its use has been banned in the European Union because it has been shown to contaminate honey and could possibly lead to the buildup of antibiotic resistance in humans [34]. A natural product that is completely safe and environmentally friendly is desirable, especially for organic beekeepers. There have been a number of recent developments in this area which include using phytochemicals, Bee Cleanse, zeolite clinoptilolite, plant extracts, and propolis extract that have been documented to be effective alternative treatments [30,35,36,37,38]. These studies generally show lowered parasite loads and improved survival of the treated bees, but very few assess the health of the bee to determine how the survival of the treated bees are being increased. Among the various promising substances to control *N. ceranae-* infection, ApiHerb^®^ and Api-Bioxal^®^, commercial dietary supplements were used as treatments against *N. ceranae*-infection effectively in both laboratory and colony level [39]. The commercial probiotics, Vetafarm Probotic, Protexin Concentrate single-strain (*Enterococcus faecium)*, and Protexin Concentrate multi-strain (*Lactobacillus acidophilus*, *L. plantarum*, *L. rhamnosus*, *L. delbrueckii*, *Bifidobacterium bifidum*, *Streptococcus salivarius*, and *E. faecium* [40], and *Parasaccharibacter apium* (PC1 sp.) and *Bacillus* sp. (PC2 sp.) [41] also were used to reduce spore loads and mortality in *N. ceranae* infected-honey bees. Currently, the control of *N. ceranae*-infections involves the use of the natural compounds to stimulate the immunity of honey bee, *A. mellifera* by inducing resistance against pathogens. Chitosan and peptidoglycans were used to reduce *N. ceranae*-infection, and increase survivorship of *N. ceranae* infected-bees. In addition, peptidoglycan and chitosan promoted the gene expression of hymenoptaecin and defensin2 [42]. Another example of natural compounds being effective at reducing *N. ceranae* loads involves using Brassicaceae defatted seed meals (DSMs) containing antimicrobial and antioxidant properties [43]. Besides fumagillin, sulforaphane was used to control *N. ceranae*-infection in laboratory. It was reported that 1.25 mg/mL of sulforaphane showed 100% reduction of spore counts, but also caused 100% bee mortality. The antimicrobial properties of this new alternative treatment may be promising, however reducing its toxicity is required before it can be considered as an alternative treatment for controlling *N. ceranae* [44].

Propolis extract of stingless bees is emerging to be an effective treatment to control *N. ceranae* across three of the four honey bee species, *A. cerana*, *A. mellifera*, and *A. florea*, [30,45,46,47]. Propolis is collected by bees and contains a number of plant resins, which are considered to be a natural product. In general, the plant resins are known to have antimicrobial effects and are used by bees to aid in sanitizing their hives. These plant resins also have recently been found to have a potential inhibitory effect on microsporidian development [30,45,46,47,48]. However, the propolis has to be fed to the honey bee in order to observe a reduction in the proliferation of *N. ceranae* in the midgut cells as the bees do not preferentially consume food containing propolis when infected. When fed, propolis extract treatment significantly enhances bee survival [30,45,46,47]. Another natural product, chito-oligosaccharides (COS) promotes antimicrobial activity and has been shown to stimulate the immune system thereby reducing *N. apis* infection in *A. mellifera* [48,49,50,51]. COS is a derivative of chitosan which is known as a biopolymer and polysaccharide found in the exoskeleton of insects and crustaceans. This water-soluble glycoprotein molecule has been used as a pre-biotic for gastrointestinal infections and diarrhea. COS is also known to aid in increased amino acid absorption across the gut lining, and also promote gut health including anti-inflammation activity through activation of 5’ AMP-activated protein kinase (AMPK) [52,53,54,55]. We, therefore, hypothesize that this treatment can aid in treating the symptoms of a *N. ceranae* infection and consequently improve the health of the honey bee.

Whether the pathological effects from a *N. ceranae* infection is of the same magnitude across the honey bee species and can be generalized to the giant honey bee, *A. dorsata*, remains unknown. *A. dorsata* serves as a main pollinator for crop plants in Thailand and provides a substantial amount of honey for a number of Asian countries [6]. Thus, the first aim of this study is to investigate the pathological effects of a *N. ceranae* infection in *A. dorsata*. Secondly, we aim to determine the efficacy of propolis extract and COS, as alternative treatment options for *N. ceranae* infections, by measuring hemolymph trehalose levels, protein contents in the hypopharyngeal gland, survival rates and acini diameters of the hypopharyngeal glands as health status indicators.

## 2. Results

### 2.1. Hemolymph Trehalose Levels

**Figure 1:**
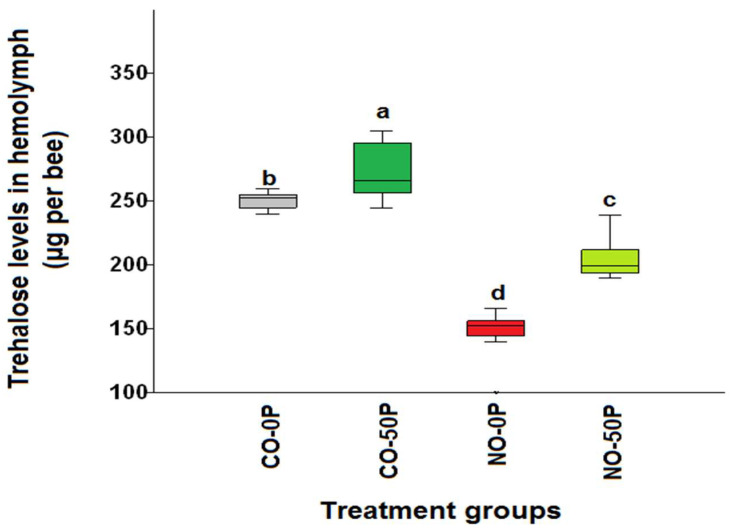
A box plot with the lines representing the median levels of hemolymph trehalose across the treatment groups of the propolis extract experiment. The control bees (CO-0P) (grey), propolis control bees (CO-50P) (green), *N. ceranae*-infected bees not treated with propolis extract (NO-0P) (red) and infected bees treated with 50% propolis extract (NO-50P) (light green) are represented by each box plot. The hemolymph trehalose levels are measured on 14 days p. i. The box indicates the inter-quartile range while the vertical bars indicate the range of the data. The different letters above each box represent significant differences (Kruskal–Wallis test: *χ*^2^ = 34.52, df = 3, *p <* 0.0001).

**Figure 2:**
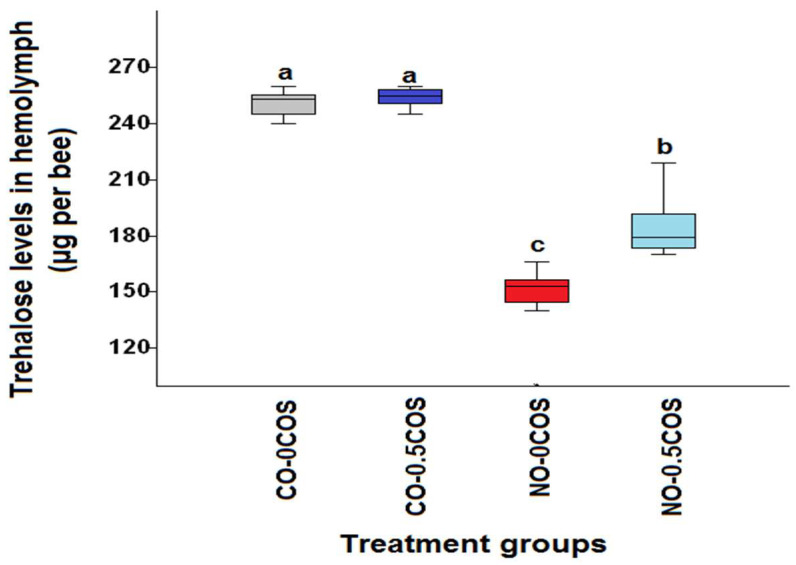
A box plot showing the median hemolymph trehalose levels data across treatments from the COS experiment. The control bees not treated (CO-0COS) (grey), the treated COS control bees (CO-0.5COS) (purple), *N. ceranae*-infected bees not treated (NO-0COS) (red) and the infected bees treated with 0.5 ppm (NO-0.5COS) (light blue) are each indicated by a box plot. The hemolymph trehalose levels were measured on day 14 p.i.. The boxes indicate the interquartile range, while the vertical bars represent the range of the data. The different letters above each box plot represent significant differences (Kruskal–Wallis test: *χ*^2^ = 33.21, df = 3, *p* < 0.0001).

*N. ceranae*-infected bees without any treatment had the lowest hemolymph trehalose levels on day 14 p.i. compared to all other treatment groups (*χ*^2^ = 34.52, df = 3, *p* < 0.0001, Figure 1). The highest levels of hemolymph trehalose were found in uninfected bees treated with propolis extract, CO-50P (273.2 ± 6.69 μg/bee) followed by the control group, CO-0P (250.8 ± 2.26 μg/bee). However, *N. ceranae*-infected bees treated with 50% propolis extract (NO-50P) showed higher levels of trehalose (204.2 ± 5.13 μg/bee) than that of *N. ceranae*-infected bees without propolis extract treatment, NO-0P (148.0 ± 5.79 μg/bee). Interestingly, similar trend was found in bees treated with COS where the highest hemolymph trehalose levels were found in the control group with 0.5 COS (CO-0.5COS) and without COS(CO-0COS) treatment 250.8 ± 2.26 μg/bee and 254.2 ± 1.73 µg/bee, respectively. The lowest hemolymph trehalose levels were found in the *Nosema* infected bees without treatment (NO-0COS) 148.0 ± 5.79 µg/bee, while there was a significant increase in the infected bees that received a COS treatment (NO-0.5COS) 184.2 ± 5.14 μg/bee (*χ*^2^ = 33.21, df = 3, *p* < 0.0001, Figure 2).

### 2.2. Hypopharyngeal Gland Protein Content

**Figure 3:**
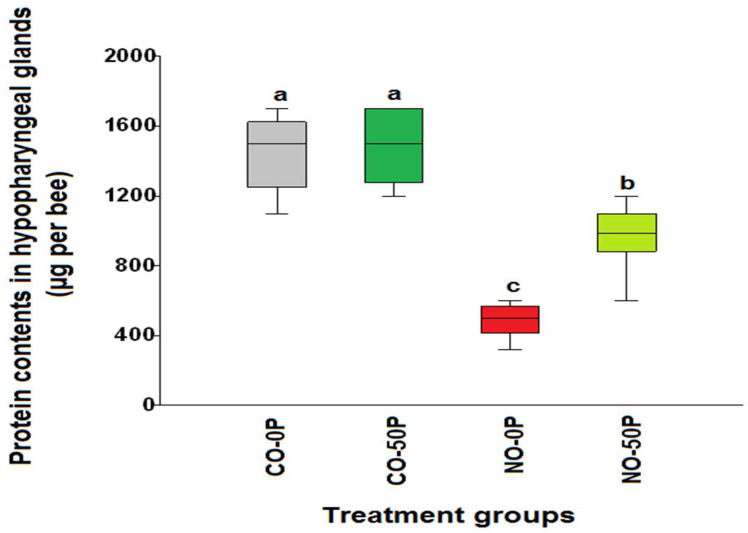
A box plot showing the median levels of hypopharyngeal gland protein contents across the treatments from the propolis extract experiment: control bees (CO-0P) (grey), control bees treated with propolis extract (CO-50P) (green), *N. ceranae*-infected bees not treated with propolis (NO-0P) (red) and infected bees treated with 50% propolis extract (NO-50P) (light green). The hypopharyngeal gland protein content was measured 14 days p.i. The boxes indicate the interquartile range, while the vertical bars indicate the range of the data. The different letters above each box plot represent significant differences (Kruskal–Wallis test: *χ*^2^= 31.75, df = 3, *p* < 0.0001).

**Figure 4:**
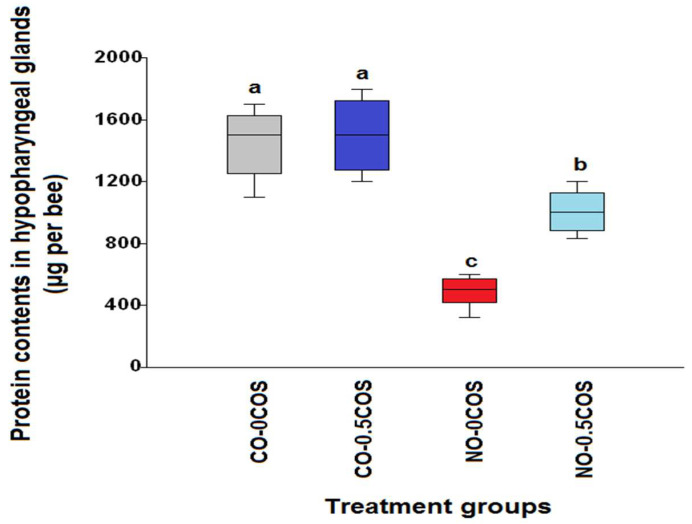
A box plot showing the median hypopharyngeal gland protein content levels from the COS experiment. The following treatments are shown with each box plot: control bees not treated with COS (CO-0COS) (grey), control bees treated with 0.5 ppm COS (CO-0.5COS) (purple), *N. ceranae*-infected bees not treated with COS (NO-0COS) (red) and infected bees treated with 0.5 ppm of COS (NO-0.5COS) (light blue). The hypopharyngeal gland was measured on day 14 p.i. The boxes represent interquartile ranges, while the vertical bars indicate the range of the data. The different letters above the box plots represent significant differences (Kruskal–Wallis test: *χ^2^*= 31.39, df = 3, *p* < 0.0001).

The hypopharyngeal gland protein contents of control bees treated with propolis extract (CO-50P) and not treated with 50% propolis extract (CO-0P) were significantly higher than the infected bees (*χ*^2^ = 31.75, df = 3, *p* < 0.0001, Figure 3), they were 1470.0 ± 65.06 μg/bee and 1326.23 ± 103.4 μg/bee, respectively. The *N. ceranae*-infected bees treated with propolis extract (NO-50P) had significantly higher protein levels, 963.0 ± 52.77 μg/bee, in comparison to the infected bees not treated with propolis extract (NO-0P), which had 486.0 ± 32.5 μg/bee of protein, respectively (*χ*^2^ = 31.75, df = 1, *p* = 0.0002). The similar trend was found in both groups of bees treated with 0.5COS (*χ*^2^ = 31.39, df= 3, *p* < 0.0001, Figure 4). The highest protein content was found in the control bees treated with COS (CO-0.5COS) (1500.0 ± 76.01 µg/bee), and it was not significantly different from that of control bees not treated with COS (CO-0COS) (*χ*^2^ = 31.39, df= 1, *p* = 0.5657). However, infected bees treated with 0.5 ppm COS had significantly increased protein content of the hypopharyngeal glands (1006.0 ± 44.4 µg/bee), in comparison with *N. ceranae*-infected bees that were not treated with COS (486.0 ± 32.5 µg/bee).

### 2.3. Acini Diameters of Hypopharyngeal Glands

**Figure 5:**
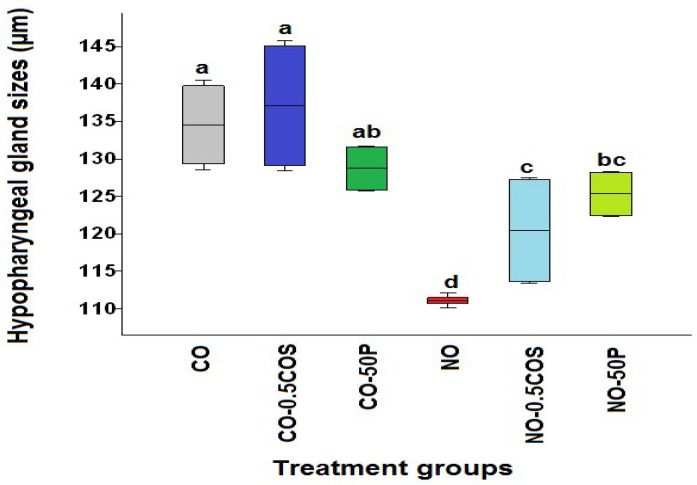
A box plot showing the median acini diameters across the treatments for the propolis extract and COS experiments. Each box plot represents a treatment: *A. dorsata* infected with *N. ceranae* dosages 10^6^ spores per bee without any treatment (NO) (red), *N. ceranae*-infected bees treated with propolis extract (NO-50P) (light green), *N. ceranae*-infected bees treated with 0.5 ppm COS (NO-0.5COS) (light blue), control bees without any treatment (CO) (grey), control bees treated with 0.5 ppm COS (CO-0.5COS) (purple), and control bees treated with 50% propolis extract (CO-50P) (green). The boxes indicate interquartile ranges, while the vertical bars represent the range of the data. The different letters above each box plot represents significant differences (Kruskal–Wallis test: *χ*^2^ = 33.09, df = 5, *p <* 0.0001).

The smallest acini diameter on average was found in the *Nosema*-infected workers (NO) without any treatment, with a distance of 111.05 ± 0.4 µm. The mean diameters of acini of the hypopharyngeal glands were largest in the untreated control bees (CO) (134.55 ± 5.22 µm) and the COS treated control bees (CO-0.5COS) (137.13 ± 8.73 µm), followed by the control bees treated with propolis extract (CO-50P) (128.75 ± 2.9 µm), the infected bees treated with the propolis extract (NO-50P) (125.34 ± 2.9 µm), and the infected bees treated with COS (NO-0.5COS) (120.44 ± 6.8 µm). When we compare between *Nosema*-infected bees and the ones treated with propolis extract and COS, we see a significant increase in the acini diameter on average (*χ*^2^ = 33.09, df = 5, *p* < 0.0001, Figure 5). However, the COS treated bees have significantly lower acini distances than the control bees (*χ*^2^ = 33.09, df = 1, *p =* 0.0022), but the propolis extract treated bees do not have a significant difference in acini distance in comparison to the control bees treated with propolis (*χ*^2^ = 33.09, df = 1, *p =* 0.0553).

**Figure 6:**
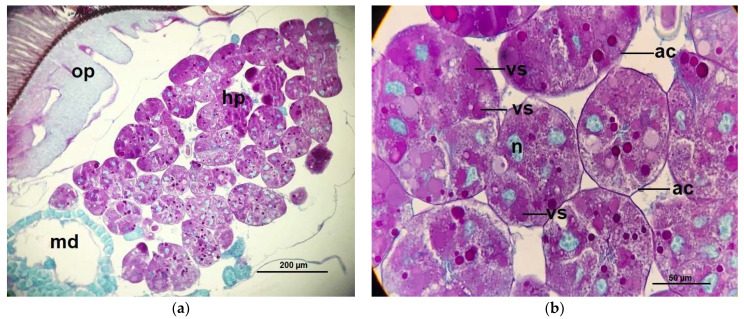
Histology cross sections of the hypopharyngeal gland of *A. dorsata* worker: (**a**) the completely developed secretory units of the glands on 14 dpi of control bees (CO). The secretory cells contain secretory granules surrounded the large nuclei of the secretory cells; (**b**) a section of the hypopharyngeal gland of CO bees with the high magnification of light microscope, the cytoplasm of the secretory cell is seen to contain variable numbers of secretory vesicles (stained red-pink with PAS). The oval nuclei are stained greenish with light green. Abbreviations: ac, acinus; hp, hypopharyngeal gland; md, mandibular gland; n, nucleus; op, optic lobe; vs, secretory vesicle.

**Figure 7:**
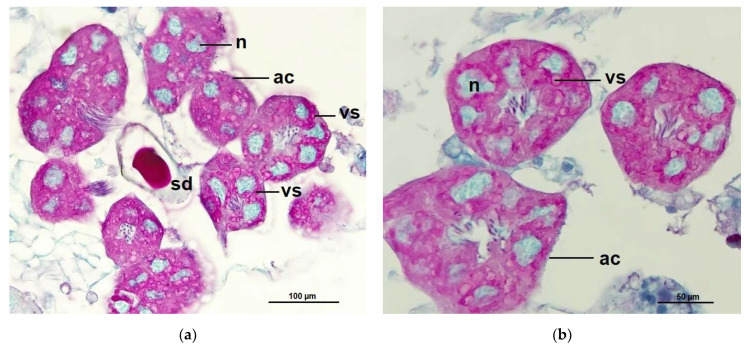
A section of the hypopharyngeal gland of 10^6^ *N. ceranae*-infected *A. dorsata* worker (**a**) on 14 dpi of 10^6^ *N. ceranae*-infected bees (NO), the cell cytoplasm contains variable numbers of secretory granules stained red-pink with PAS. The large oval loose nuclei are stained greenish from a light green dye used as a counterstain; (**b**) A medial section of NO bees on 14 dpi, the secretory cell contains secretory granules surround the large nuclei of the secretory cells. Abbreviations: ac, acinus; n, nucleus; sd, secretory duct; vs, secretory vesicle.

**Figure 8:**
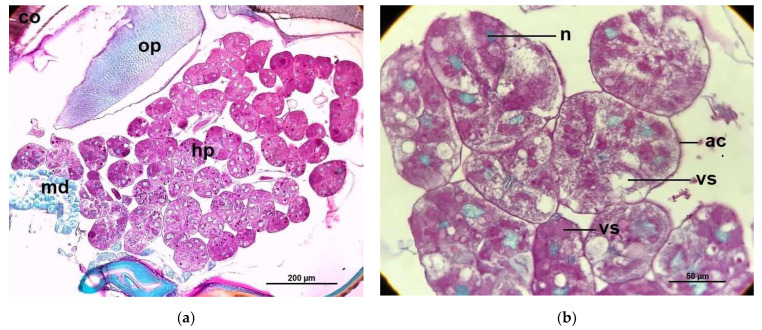
The light micrographs of: (**a**) A section of the hypopharyngeal glands of 10^6^ *N. ceranae*-infected bees on14 dpi, treated with 50% propolis (NO-50P). The cytoplasm of the secretory cell contains variable numbers of secretory granules stained red-pink with PAS. The oval nuclei are stained a greenish color from light green; (**b**) with higher magnification of NO-50P shows the secretory cell contains several secretory vesicles with negative staining using PAS, and also contains secretory vesicles with smaller amounts of carbohydrate, which are characterized by a red-pink color from PAS staining. Abbreviations: ac, acinus; co, compound eyes; hp, hypopharyngeal gland; md, mandibular gland; n, nucleus; op, optic lobe; vs, secretory vesicle.

**Figure 9:**
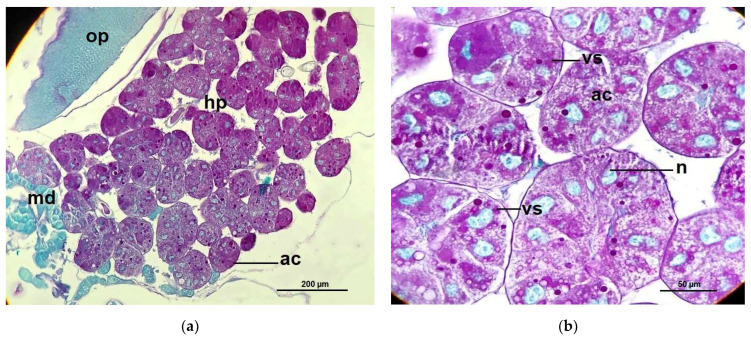
(**a**) A cross section of the hypopharyngeal gland from 10^6^ *N. ceranae*-infected *A. dorsata* bees on 14 dpi that were treated with 0.5 ppm COS (NO-0.5COS). The cytoplasm of the secretory cells contains variable numbers of secretory granules stained red-pink with PAS. The oval nuclei are stained greenish from a light green; (**b**) a medial cross section of the hypopharyngeal gland from 10^6^ *N. ceranae*-infected *A. dorsata* bees on 14 dpi that were treated with 0.5 ppm COS (NO-0.5COS). Abbreviations: ac, acinus; hp, hypopharyngeal gland; md, mandibular gland; n, nucleus; op, optic lobe; vs, secretory vesicle.

The histological structure of the hypopharyngeal glands of CO bees showed fully developed and contained with several secretory units or acini (oval to rounded shape), each unit composed of 5–8 secretory cells surrounded a central secretory duct. The secretory cells contained with numerous secretory granules stained red-pink with PAS that surround the large cell nuclei, stained greenish with light green (Figure 6), while the secretory units of the hypopharyngeal glands of *Nosema*-infected bees (NO) were incomplete developed in structure indicated by different irregular in shaped and sizes. Therefore, each cell cytoplasm consisted of numerous small secretory vesicles stained pink with PAS (Figure 7). Interestingly, the glands of NO-50P and NO-0.5COS showed fully developed acini. The secretory cell contains several vesicles giving both positive and negative staining with PAS, this indicated the cell storage both carbohydrate and non-carbohydrate molecules. In addition, the large extracellular space between adjacent acinar cells were found indicated by white gap between adjacent cells separating them from each other (Figure 8 and Figure 9).

### 2.4. Honey Bee Survival Rates

**Figure 10:**
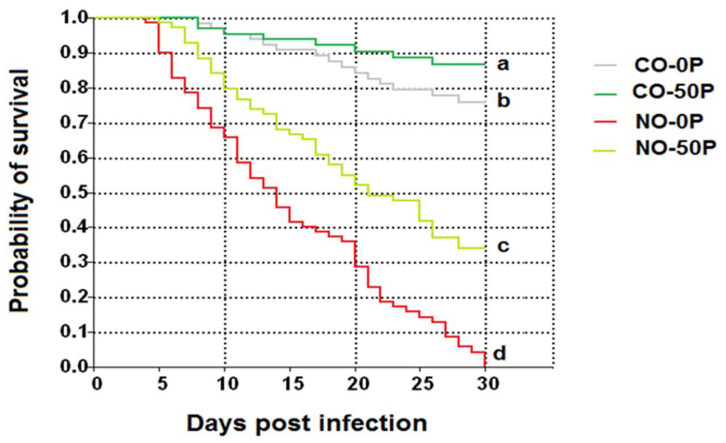
Kaplan–Meier survivorship curves of *A. dorsata* workers after being infected with 10^6^ *N. ceranae* spores (NO-0P), versus infected bees that received a propolis treatment (NO-50P) or no infection and a propolis treatment (control: CO-0P and CO-50P). Survivorship curves with different letters within treatments are significantly different (Kruskal–Wallis test: *χ*^2^ = 17.33, df = 3, *p =* 0.0005).

**Figure 11:**
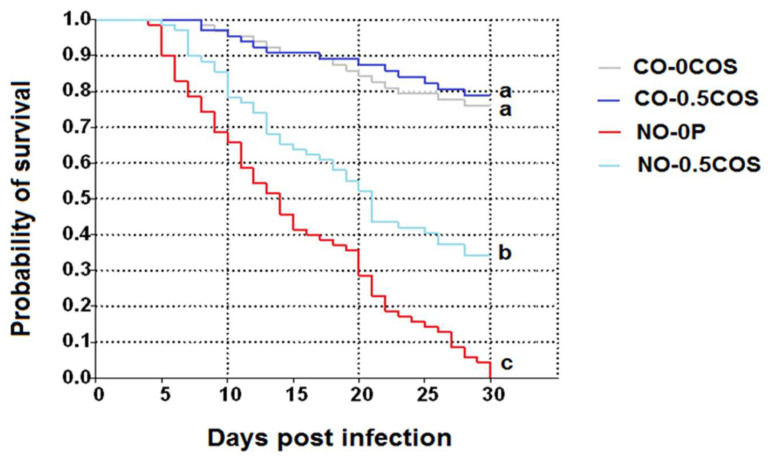
Kaplan–Meier survivorship curves of *A. dorsata* workers after *N. ceranae* infection at 10^6^ spores (NO-0COS), NO-0.5COS or no infection (control: CO-0COS and CO-0.5COS). Survivorship curves with different letters within treatments are significantly different (Kruskal–Wallis test: *χ*^2^ = 16.08, df = 3, *p =* 0.0010).

Kaplan–Meier curves showed that *A. dorsata* workers infected with *N. ceranae* dosed with 10^6^ spores per bee (NO-0P) had significantly lower survival in comparison to the infected bees that received propolis treatment (*χ*^2^ = 17.33, df = 3, *p =* 0.0005, Figure 10). The control bees treated with propolis extract (CO-50P) had the highest survival, followed by CO-0P and NO-50P, respectively. A similar trend was found in bees treated with 0.5 ppm COS, except in this case there was no significant difference between the control bees and the control bees treated with COS (*χ*^2^ = 16.08, df = 3, *p =* 0.0010, Figure 11).

## 3. Discussion

The increase of hemolymph trehalose levels, protein content of the hypopharyngeal glands, and enhanced acini diameters of hypopharyngeal glands of the infected giant honey bee, *A. dorsata*, all indicate improved health after 50% propolis extract and 0.5 ppm COS treatment. Although the levels after treatment were not to the same level of the uninfected control bees, except for the acini diameter from the propolis treatment, there was still a significant increase for all health measures in comparison to the infected bees without any treatment. Based on our results, both stingless bee propolis extract and COS are effective treatments in improving the health of the honey bee. Propolis extract however may be a slightly better treatment as indicated by the full recovery of the acini diameter distance of the hypopharyngeal gland, which was not the case for the COS treatment. For both treatments there were no detrimental effects in the uninfected control bees for hemolymph trehalose levels, protein content in the hypopharyngeal gland, and the acini diameters in the hypopharyngeal gland, which suggests that these treatments are not having any damaging side-effects for these health parameters measured. Whether the increased trehalose levels and protein content of the hypopharyngeal gland is due to a lower parasite load or the improved nutrient absorption across the gut lining in treated bees—which results in higher tolerance of the parasite—remains to be investigated.

The higher trehalose levels in the uninfected bees treated with propolis extract in comparison to the uninfected bees without any treatment suggests that propolis might be affecting the sugar metabolism of the honey bee. This is interesting to note because hemolymph trehalose levels are central to buffering against the energetic stress suffered from the infected bees [24]. In addition, increased trehalose levels were found to be a key difference in honey bees selected to better tolerate *N. ceranae* infections [56]. Therefore, the increased hemolymph trehalose levels from propolis consumption could be one way in which increased survival results from infected bees treated with this [30]. Propolis extract also positively increased the protein contents of hypopharyngeal glands, and the acini diameters of hypopharyngeal glands of honey bee. This suggests that the increased health measures may also be due to the lowering of the *N. ceranae* load in the treated bees. This corresponds to the results of the previous study which showed the potential of propolis extracted from stingless bee to control *N. ceranae* infection in the red dwarf honey bee, *A. florea* [30]. Recently, it has also been demonstrated that propolis extract can increase the survival of *A. mellifera* [45,46]. Taken together, these results suggest that propolis extract may have a general positive impact on bee health, across all honey bee species. Further supporting this notion is the fact that stingless bee propolis extracts are known to specifically have antifungal properties [57]. Moreover, a previous study demonstrated abnormal structure of *N. ceranae* spores, inside *A. cerana* bees, after being treated with propolis extract, which corresponded with the interference of spore growth and development [47].

Bees treated with COS after infection with *N. ceranae* also had significantly higher trehalose levels, protein contents of hypopharyngeal glands, and increased acini diameters. However, it is more likely that these effects are resulting from indirect mechanisms such as enhanced bee immunity or increased nutrient absorption across the gut lining as opposed to directly reducing the reproduction and growth of the *N. ceranae* infection. COS is known to improve nutrient digestibility, gut functions and gut modifications in animals [50]. COS may affect *Nosema* development, but is more likely to achieve this through enhancing bee immunity, that will eventually result in higher hypopharyngeal gland protein contents, trehalose levels, and the increasing hypopharyngeal gland acini from decreased *N. ceranae* loads [58]. This is plausible because *N. ceranae* typically suppresses the immune system in infected bees in order for increased growth and reproduction inside the host [17,59].

It is important to note that *N. ceranae* can infect *A. dorsata* and develop well in this host. As previously shown, *A. mellifera*, *A. cerana* and *A. florea* can also be infected by *N. ceranae*. To date, all of the honey bee species have now been shown that not only can become infected with this parasite, but they are also suffering from the pathological effects of the infections as well. Based on our results *A. dorsata* is no exception, which raises concerns as the honey bee species may be suffering from some of the same behavioral and physiological changes that have been documented in *A. mellifera* from a *N. ceranae* infection [27]. Previous results show that the parasite develops well in each of the four honey bee species and that the intracellular life cycle is completed within three days p.i. [60,61,62]. Due to the successful reproduction in all four of the honey bee species there are opportunities for cross transmission between the species on a community level as they have overlapping foraging ranges and are known to share the same floral species when foraging [62,63,64].

The reduction in acini diameter of the hypopharyngeal glands of *N. ceranae*-infected bees might due to deficiency of amino acids used for secretory cell development. This is not surprising because previously it has been shown that the amino acid profiles in the hemolymph of infected bees is altered and feeding pollen can increase the survival of infected bees [65,66]. The recovery in the hypopharyngeal gland protein is important because it has been noted to play a role in protein synthesis of royal jelly production [6]. Metabolite dysregulation of royal jelly secretions has been documented in *N. ceranae* infected bee hives, which has implications for the antibacterial effectiveness of the secretions when feeding the brood [18]. Our findings suggest that COS and propolis extract treatment is likely to contribute to the increase of royal jelly productivity as well at the colony level, due to the increase of protein contents and the acini diameters of the hypopharyngeal glands. The lowering of the immune system is likely to be a result in the lack of protein nutrition resulting from the force feeding of spores that geminate and proliferate within the midgut epithelial cells where they disrupt host nutrient absorption [67,68]. Although infected bees do not exhibit obvious external disease symptoms, some of the main pathophysiological effects from an infection identified from omics studies have pointed to metabolic dysregulation [18,21,25,69]. Resulting from this metabolic dysregulation are the key symptoms of infection, which are lowered trehalose levels and reduced hypopharyngeal glands [22,24,62]. Therefore, we find that the measures used in this study to be accurate predictors of bee health and recovery from a *N. ceranae* infection. The effects of a *N. ceranae* infection on the colony level include lower colony population and the reduction of honey production [61,70]. Thus, we are interested if this treatment at the colony level might show an improvement in these colony level symptoms of infection.

The use of natural product such as propolis from stingless bees and COS will facilitate new strategies that can be used to control *Nosema* and improve honey bee health and beekeeping production. However, further experiments could be performed to determine the optimal doses to maximize the effect for each of the treatments. In addition, long term treatments could be investigated on a colony level to determine if they are great enough to effectively reduce the *N. ceranae* parasitic loads in a more natural setting. Perhaps using both treatments at the same time will synergistically improve the overall effectiveness in improving the health and survival of the honey bee. On one hand, *N. bombi* might also be another suitable target for this treatment which could lead to the health improvement of bumble bees as well, but on the other hand, the non-lethal side effects of these treatments should be investigated to determine if they pose any detriment to the health of bees. All of these would be interesting avenues to pursue in the future to further understand the practical use of stingless bee propolis extract and COS in terms of managing *N. ceranae* infections around the world.

## 4. Materials and Methods

### 4.1. Propolis Extraction

We collected propolis from three different stingless bee, *Tetrigona apicalis*, colonies from an apiary located in Chanthaburi Province, Thailand. We then dried the propolis in a hot air oven (Binder ED 53, BINDER GmbH, Tuttlingen, Germany) at 80 °C for 72 h, this was then frozen at −21 °C (Sharp SJ-X43T, Sharp Thai Co., Ltd. (STCL), Bangkok, Thailand) for 3 h and grinded using a motor and pestle. We extracted 60 g of propolis powder with 100 mL of 70% ethanol for 72 h, this was then followed by gravity filtration using a Whatman No. 4 filter paper [30]. After filtration a crude ethanol extract was formed that we defined as 100% propolis stock solution. For the experiments a 50% propolis solution was prepared by diluting the stock solution with water (*v/v*).

### 4.2. Chito-Oligosaccharide Solution Preparation

We made a 10^4^ ppm stock of COS, by taking 0.25 g of COS (6081 Da) and dissolving it in 5 mL of pure *A. dorsata* honey (pH = 3.45). We then adjusted the final volume of this to 20 mL with 50% sucrose solution (*v/v*). We then diluted the 10^4^ ppm stock solution of COS to a 50% honey solution using water (*v/v*) to make a final concentration of 10^2^ ppm. Afterwards we then prepared a 0.5 ppm COS solution using the same methods that had a pH of 3.77 (pH meter, Mettler Toledo Gmbh, Greifensee, Zurich, Switzerland).

### 4.3. Spore Preparation

*Nosema ceranae* spores were propagated from heavily infected *A. florea* colonies located in the Chon Buri Province of Thailand. We fed isolated spores to *A. mellifera* workers (5 × 10^7^ spores for 50 bees) that were kept at 34 ± 2 °C (Memmert IPP 260, Schwabach, Germany) with relative humidity (Barigo-8861, Schwenningen, Germany) (RH) between 50–55% for 14 days in order to propagate more spores for the experimental infections. To propagate more spores, midguts were removed and transferred to a 1.5 mL microcentrifuge tube containing 100 µL distilled water. The midguts were then homogenized using a sterile pestle and centrifuged at 6000× *g* (Benchmark Scientific Z206-A, Sayreville, NJ, USA) for 10 min, this was repeated for 3 times [40]. We discarded the supernatant each time and the white sediment at the bottom was collected to be counted using a hemocytometer (Hausser Scientific, Horsham, PA, USA) under a light microscope (Olympus CX50, Shinjuku, Tokyo, Japan) [71]. After one more centrifugation, we re-suspended the spores in 50% (*w/v*) sucrose solution to make a final concentration of 5 × 10^5^ spores per µL. We stored this syrup at room temperature overnight until further use.

### 4.4. Propolis Extract and COS Treatment Experiments

We obtained 3 frames of sealed brood from three *Nosema* free colonies of *A. dorsata* located in Samut Songkhram Province, Thailand. Colonies were confirmed to be *Nosema* free following standard procedures [72,73]. To obtain newly emerged bees, the brood frames were kept in an incubator (Memmert IPP 260, Schwabach, Germany) at 34 ± 2 °C with RH (Barigo-8861, Schwenningen, Germany) between 50–55%. The newly emerged bees, between 24–48 h of age, were confined to cages, in groups of 50, and divided into 8 groups. The first 4 groups, were individually force-fed with 2 µL 50% sucrose solution (*v/v*) containing 10^6^ *N. ceranae* spores per bee. We then provided 2 groups with 2 mL of either 0% or 50% stingless bee propolis extracts, daily, and these groups were defined as NO-0P and NO-50P, respectively. For the other 2 groups, we provided 2 mL of 0 ppm or 0.5 ppm of COS defined as NO-0COS and NO-0.5COS, respectively.

The control groups were individually force-fed with only 50% sucrose solution (*v/v*), and were defined as the negative control bees CO-0P, CO-0COS, CO-0.5COS, and CO-50P, respectively. In the CO-50P control group was each bee was also treated daily with 2 mL of 50% stingless bee propolis extract, while in the CO-0.5COS control group the bees were treated daily with 2 mL of 0.5 ppm COS. For the duration of the experiment, each cage was fitted with two gravity feeders, one containing distilled water, and the other sugar syrup (50% *w/v* sucrose solution). We also supplied 60 g of pollen mixed with 17 mL of 50% sucrose solution (*w/v*), each was replenished as necessary throughout the experiment. All cages were placed in an incubator at 34 ± 2 °C (Memmert IPP 260, Schwabach, Germany), with a RH ranging from 50–55%. The 50% stingless bee propolis extract was provided in 2 mL at a time in a 1.5 mL micro-centrifuge tube from the start of the experiment (0 Day p.i.), until the end (30 Days p.i.), and was replaced as necessary. For the COS treatment, we provided 2 mL of 0.5 ppm COS in 50% honey solution in a 1.5 mL micro-centrifuge at the start of the experiment (0 Day p.i.) and this was replaced as necessary until the end of the experiment (30 Days p.i.).

### 4.5. Hemolymph Trehalose Measurements

On Day 14 p.i., 10 honey bees were removed from each cage and were anaesthetized at −21 °C for 5 min. Before we collected their hemolymph, honey bees were mounted on a wax plate by a pair of insect pins crossing over the waist. Using a glass microcapillary (Hirschmann^®^ Laborgerate, Eberstadt, Germany), 5 µL per bee was collected by puncturing abdomen segments between tergites 3 and 4, and the hemolymph was transferred to a microcentrifuge tube (Eppendorf, Hamburg, Germany) containing 45 µL of 0.85% NaCl. For each sample, 2.9 mL of anthrone reagent was added and then vortexed for 30 s before we quickly put them into a boiling water bath for 15 min. After this they were placed into cold water (4 °C) for 20 min and read at 620 nm absorbance using a Shimadzu UV-visible spectrophotometer (UV-1610). Quantification of the hemolymph trehalose amounts were based on a standard curve.

### 4.6. Hypopharyngeal Gland Protein Content Measurements

Another 10 bees were randomly removed from each cage at 14 days post infection (p.i.). These bees were decapitated so that their hypopharyngeal glands could be removed under a stereomicroscope (Olympus CH30, Shinjuku, Tokyo, Japan). Glands of each bee were stored in 50 µL of phosphate buffer solution (pH 7.8) in a 1.5 mL microcentrifuge tube. These were then homogenized and centrifuged at 1000× *g* for 2 min. Supernatant from each tube was used in the Bradford protein assay [74]. Quantification of protein content was based on standard curves that were prepared using bovine serum albumin (BSA). Protein absorbance was measured at 595 nm absorbance against a blank reagent using a Shimadzu UV-visible spectrophotometer (UV-1610).

### 4.7. Measurements of Acinar Sizes of the Hypopharyngeal Glands and Histological Structure

Another 10 bees from each group were collected on 14 days p.i. and the heads were dissected in insect saline (NaCl 7.5 g/L, Na_2_HPO_4_ 2.38 g/L, KH_2_PO_4_ 2.72 g/L) and then fixed in Bouin’s solution for 24 h. Samples were dehydrated using a series of increasing ethyl alcohol concentrations: 70%, 90%, 95%, and 100% for 10 min per concentration. Samples were then soaked in xylene for 1 h and then embedded in paraffin wax. The tissues were sectioned into 6 µm thickness using a rotary microtome (Leica, Wetzlar, Germany), and then stained with Periodic acid Schiff’s reagent (PAS) followed by a counter staining of light green dye [75,76]. Measurement of acinar sizes of the hypopharyngeal glands were made under a light microscopy (Olympus CX 50, Shinjuku, Tokyo, Japan) using a micrometer (ERMA: ESM-11, Japan); *n* = 10 per bee each treatment.

### 4.8. Survival Analysis

Survivorship curves of all treatment groups were generated using the Kaplan–Meier approach by plotting number of surviving bees against days from initiation of the experiment [30]. Honey bee survival rates were compared across the treatment groups using a non-parametric, univariate analysis of variance and a corresponding post hoc test (Kruskal–Wallis test and the Mann–Whitney U test).

### 4.9. Statistical Analyses

Hemolymph trehalose levels, protein contents of the hypopharyngeal gland, the diameter of the hypopharyngeal gland acini of *N. ceranae*-infected bees on day 14 p.i., and the survival rates were normally distributed (Jarque–Bera JB test: *p >* 0.05) but had unequal variances (Levene’s test: *p <* 0.05). We, therefore, used a non-parametric Kruskal–Wallis test and a Mann–Whitney U test to compare across the treatment groups. Multiple comparisons were accounted for using a Bonferroni correction.

## Data Availability

The data presented in this study are available on requested from the corresponding author.
